# Coral Settlement on a Highly Disturbed Equatorial Reef System

**DOI:** 10.1371/journal.pone.0127874

**Published:** 2015-05-20

**Authors:** Andrew G. Bauman, James R. Guest, Glenn Dunshea, Jeffery Low, Peter A. Todd, Peter D. Steinberg

**Affiliations:** 1 Advanced Environmental Biotechnology Centre, Nanyang Environment and Water Research Institute, Nanyang Technological University, Singapore, Singapore; 2 School of Biological, Earth and Environmental Science and Centre for Marine Bio-Innovation, University of New South Wales, Sydney, New South Wales, Australia; 3 Ecological Marine Services, Burnett Heads, Queensland, Australia; 4 National Biodiversity Centre, National Parks Board, Singapore, Singapore; 5 Experimental Marine Ecology Laboratory, Department of Biological Science, National University of Singapore, Singapore, Singapore; Leibniz Center for Tropical Marine Ecology, GERMANY

## Abstract

Processes occurring early in the life stages of corals can greatly influence the demography of coral populations, and successful settlement of coral larvae that leads to recruitment is a critical life history stage for coral reef ecosystems. Although corals in Singapore persist in one the world’s most anthropogenically impacted reef systems, our understanding of the role of coral settlement in the persistence of coral communities in Singapore remains limited. Spatial and temporal patterns of coral settlement were examined at 7 sites in the southern islands of Singapore, using settlement tiles deployed and collected every 3 months from 2011 to 2013. Settlement occurred year round, but varied significantly across time and space. Annual coral settlement was low (~54.72 spat m^-2^ yr^-1^) relative to other equatorial regions, but there was evidence of temporal variation in settlement rates. Peak settlement occurred between March–May and September–November, coinciding with annual coral spawning periods (March–April and October), while the lowest settlement occurred from December–February during the northeast monsoon. A period of high settlement was also observed between June and August in the first year (2011/12), possibly due to some species spawning outside predicted spawning periods, larvae settling from other locations or extended larval settlement competency periods. Settlement rates varied significantly among sites, but spatial variation was relatively consistent between years, suggesting the strong effects of local coral assemblages or environmental conditions. Pocilloporidae were the most abundant coral spat (83.6%), while Poritidae comprised only 6% of the spat, and Acroporidae <1%. Other, unidentifiable families represented 10% of the coral spat. These results indicate that current settlement patterns are reinforcing the local adult assemblage structure (‘others’; i.e. sediment-tolerant coral taxa) in Singapore, but that the replenishment capacity of Singapore’s reefs appears relatively constrained, which could lead to less resilient reefs.

## Introduction

Scleractinian corals, the key ecosystem engineers of tropical coral reefs [1], face severe threats in many coral reef regions [2–4]. The combined impacts of multiple anthropogenic disturbances (i.e. overfishing, pollution and sedimentation), coupled with the more recent superimposed effects of climate change, have caused widespread coral mortality and recruitment failure [5,6] resulting in many reefs being unable to recover from additional perturbations [6,7]. On some reefs this has led to strong directional shifts in the taxonomic structure of coral communities [8, 9], while on other reefs dramatic transitions from dominance by corals to dominance by fleshy macro-algae [10,11] or other non-coral organisms [12] have occurred. The dramatic changes occurring in coral reef ecosystems have precipitated a need to better understand early life history process critical for the replenishment of coral populations [6,13].

Population replenishment is an important demographic process for the persistence of all marine organisms with open populations [14,15]. For coral reefs, a key element in their maintenance, recovery and resilience is the successful re-establishment or recruitment of coral functional groups characteristic of the locality [6,16]. While regrowth of remnant coral colonies or fragments can also be important [17], sexual recruitment provides the only means of restoring both coral cover and genetic diversity [18]. Successful coral recruitment, however, is highly dependent on many sequential early life history processes, including fecundity (e.g. [19]), fertilization rates (e.g. [20]), larval survivorship and dispersal (e.g. [21,22]), settlement (e.g. [23]), and early post-settlement growth and survivorship (e.g. [24]).

Environmental stressors that disrupt early life processes can compromise coral recruitment and profoundly affect overall coral population dynamics [25–27]. Early life history stages can thus represent a demographic bottleneck (i.e., low numbers of individuals at particular life stages) in the life cycle of corals [27]. Demographic bottlenecks may first occur pre-settlement (e.g. limited larval supply: [5]) then during settlement (e.g. space limitation: [13]) and finally during the early post-settlement period (e.g. high mortality: [27]). All of these processes are particularly sensitive to natural and anthropogenic stressors, including those caused by: thermal stress (e.g. [28,29]), increased sedimentation and turbidity (e.g. [30,31]), eutrophication (e.g. [32]), fouling (e.g. [33]) or coral bleaching (e.g. [34]). Thus knowledge of spatial and temporal patterns of early life history stages is often a prerequisite to understanding how well coral assemblages respond to environmental stress and anthropogenic changes in their environment [35].

Considerable attention has been paid to elucidating spatial and temporal variation in coral settlement at different scales, the mechanisms underlying such variation (i.e., interacting biological and physical processes), and the role of settlement in structuring adult populations [13,19,36]. The density of coral settlers can vary over months, seasons and years (e.g. [37–39]), and at multiple spatial scales, including: the upper and lower surface of settlement tiles (e.g. [40]), among tiles within a site, sites within a habitat, habitats on a reef, and reefs within a region (e.g. [23, 37, 41]). Furthermore, dominant coral genera recruiting can vary among seasons, years, and also between regions (e.g. [38,42,43]). Despite the recognized importance of understanding settlement patterns, there remains limited information regarding the importance of these ecological processes on highly urbanized and frequently impacted Indo-Pacific reefs [44].

Coral reefs in Singapore offer a unique opportunity to investigate coral settlement in a highly disturbed, equatorial environment. Corals in Singapore persist in a harsh environment created by chronic anthropogenic disturbances. Decades of coastal development, land reclamation and shipping activities have resulted in the release of large volumes of sediments into the surrounding marine environment [45,46]. Sedimentation rates and values for total suspended solids in Singapore exceed thresholds considered ‘optimal’ for most tropical reefs [46,47]. Eutrophication has increased 30 fold [48], furthermore, underwater light penetration on some reefs appears to have been substantially reduced. For example, light intensity (i.e. percentage of surface light intensity) at one site in 1973 was an estimated 83% and 10% at depths of 2 and 8 m respectively [49], whereas at the same site and depths in 2000 light penetration was ~19% and ~0.6% respectively [50]. Moreover, Singapore reefs are subject to stress from major thermal bleaching events [51]. Despite these adverse conditions, there exist diverse shallow coral communities [52], and while overall mean live coral has declined over the past two decades [53], live coral cover remains relatively high (~36% cover across reefs) compared to other Indo-Pacific reefs (e.g. Great Barrier Reef mean coral cover ~23% [4]).

High settlement or other aspects of the early life history of these corals may play an important role in the persistence of Singaporean coral reefs, but research on coral settlement in Singapore has been limited to a few small-scale studies at a few sites [54,55]. Dikou and van Woesik [54] examined coral settlement patterns and their relationship to local environmental conditions on the upper reef slopes of 3 sites over 2 years. Rates of coral settlement were extremely low (14 spat m^-2^ yr^-1^), and this was attributed to limited larval availability or settlement success [54]. These authors also reported significant differences in settlement rates and taxonomic composition of spat among sites. There have not, however been any systematic studies of seasonal patterns in coral settlement, or any explicit testing of spatial variation in rates of settlement relative to local abundance and composition of adult corals. Given the existing environmental conditions in Singapore, a more thorough examination of coral settlement processes to determine how and to what extent Singapore’s reefs replenish themselves is warranted. The purpose of this study was to quantify spatiotemporal variation of coral settlement among seven coral reef sites in the southern islands of Singapore. We also determined the taxonomic composition of coral spat, and explored the relationship between adult coral assemblages and coral settlement among different families.

## Materials and Methods

### Ethics Statement

A research permit for this work was granted by the Singapore government through the National Parks Board; permit NP/RP11-073.

### Study area

**Fig 1:**
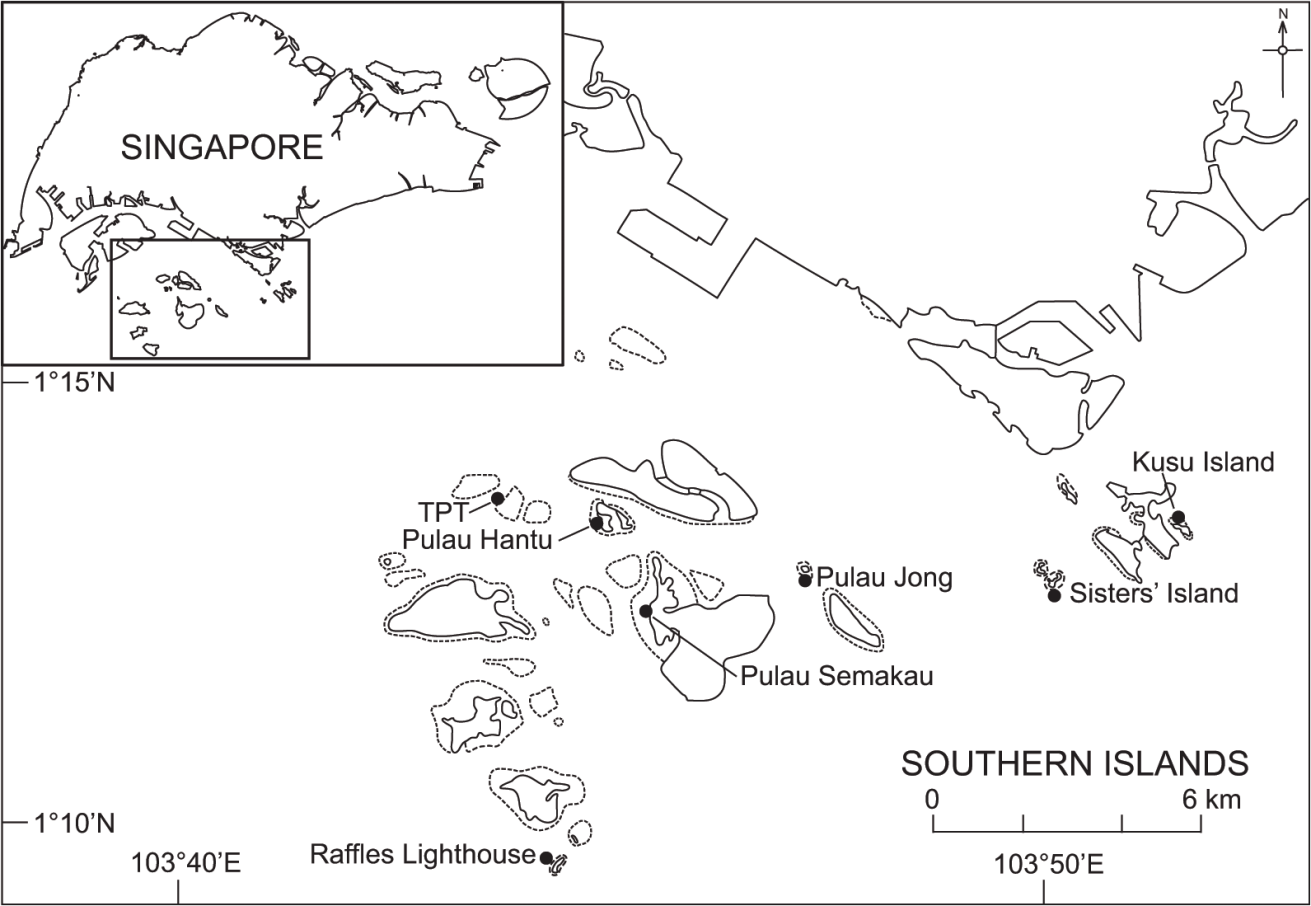
Map of southern coast of Singapore showing study sites. Sites are organized from west to east: Terumbu Pempang Tengah (TPT), Raffles Lighthouse, Pulau Hantu, Semekau, Pulau Jong, Sister’s Island and Kusu. Dotted lines represent fringing reef areas.

This study was conducted in the southern islands of Singapore, which lie ~137 km north of the equator within the Strait of Singapore (1°17’N, 103°360’E, Fig 1). Singapore consists of one relatively large main island and ~50 smaller offshore islands, the majority of which are located south of the main island (Fig 1). Most of the seafloor in this area is covered with unconsolidated sand and mud [56], making it unsuitable for coral reef development. However, there are shallow fringing coral reefs around most of the southern islands, characterized by a shore-adjacent reef flat leading seaward to the reef crest and upper reef slope down to ~8 m depth. This depth restriction is primarily due to relatively low light levels as a result of high sedimentation and siltation [45,46]. Consequently, suitable habitat for coral settlement is also restricted to these limited reef areas [54].

Seven study sites were selected across ∼15 km of coastline in southern Singapore to assess spatiotemporal variation in coral settlement (Fig 1). All sites had comparable water depths of 8–10 m to the base of the reef slope, and relatively similar exposure to environmental conditions (e.g. wind and waves). Throughout Singapore, the hydrodynamic circulation patterns are directed mainly by seasonal trade winds from the Asian monsoon cycle and by tidal forcing [57]. However, at smaller local scales (i.e. between islands) tidal forcing is considered the main driver of currents [57]. Singapore experiences a monsoonal climate, with two distinct periods, the northeast monsoon (~December to March) and southwest monsoon (~June to September). The monsoon periods are separated by two relatively shorter inter-monsoon periods from October to November and April to May.

### Settlement patterns

Coral settlement was quantified at each site using 20 unglazed and unconditioned terracotta tiles (10 × 10 × 1 cm). Settlement tiles were placed on the sheltered western sides of the fringing reefs at all sites. Tiles were attached directly to the substrate at each site following Mundy [40]. Tiles were deployed randomly at approximately 3–4 m depth, spaced ~1–2 m apart and were installed parallel to the reef crest. Stainless steel nuts were used to maintain each tile 15–20 mm above the substrate to create a ‘gap habitat” favored by coral larvae [40].

Settlement tiles were deployed in the field starting in September 2011, and were replaced every 3 months, for the 4 seasonally defined periods: September–November, December–February, March–May, and June–August, for 2 years (Year 1: September 2011–August 2012; Year 2: September 2012–August 2013). These periods were selected to correspond with the annual coral spawning event, which in Singapore occurs predominately between March and April [58,59]. Freshly collected tiles were immediately soaked in diluted bleach for ∼24–48 hrs to remove organic materials, and then rinsed and sundried before inspecting for coral spat. All coral spat were counted and identified on each tile using a dissecting microscope (40× magnification). The majority of coral spat recorded were single corallites <3 mm diameter. At this early stage of development, the morphology of the corallum is not sufficiently developed to allow high taxonomic resolution, and only three families (Acroporidae, Pocilloporidae, Poritidae) can be reliably distinguished [60]. All other coral spat were categorized as ‘others’. Coral spat that could not be identified because of overgrowth by other organisms (e.g. barnacles, bryozoans, sponge) or that were damaged during retrieval were listed as ‘damaged’.

### Adult cover patterns

Following the initial deployment of the settlement tiles, coral assemblages at each site were surveyed in November 2011 using point-intercept transects (PIT). At each site, 6 replicate 30 m long line transects were surveyed at approximately the same depth (~3–4 m) as the settlement tiles. A total of 61 points were surveyed on each transect, spaced at 0.5 m intervals for a total of 366 points site^-1^. Any scleractinian corals underlying each point were identified to genus. Percent coral cover was then calculated by taking the mean proportion of the total points for each coral genus.

### Data analysis

The primary aim of the analysis was to test whether variation in counts of coral spat on tiles could be attributed to site, sampling period and year, or any additive or interactive combination of these. To do this, coral settlement data were analyzed using generalized linear models (GLMs) for count data under a multiple working hypothesis framework [61]. This involved comparing candidate models with different combinations of predictor variables using information theoretic and classical model comparison and selecting the most parsimonious model. The response variable was counts of spat tile^-1^ and explanatory variables were site, sampling period and year (all as fixed factors). Full models, including all interactions, were fit to data and backward model selection procedures applied. GLMs with Poisson and negative binomial variance structures were initially compared for relative goodness of fit with Akaike’s Information Criteria (AIC), and the best GLM variance structure used for further backward model selection. When models were similar (i.e. had relatively close AIC values ≈ 2), nested models were compared with hypothesis tests based on model deviance (likelihood ratio tests dependent on the model error structure: [62]). If there was no significant difference between similar models, the simpler model was selected. In cases where likelihood ratio tests of different nested models had a P value within 0.02 (< or >), the simpler model was selected as a conservative approach, given that such likelihood ratio tests are approximate [62]. Selected final models were validated by visual inspection of residual plots, normal QQ plots and residuals/leverage plots. Models were interpreted by a combination of plotting predicted values at relevant spatial and temporal resolution given the final model terms and wald-tests of individual coefficients where the reference level of each factor was changed sequentially. The overall significance of terms in the final model was evaluated using likelihood ratio tests comparing nested models with and without the term.

Over half (53%) of the retrieved tiles contained no spat and only 27% of retrieved tiles contained non-Pocilloporid spat. Due to the excess of zeros for the non-Pocilloporidae spat counts, zero-inflated models with Poisson and negative binomial count variance structures were initially compared. Zero-inflated models account for excess zeros in sample data by modelling zeros as a mixture arising from a binomial and a count process. The binomial process divides the data into a ‘zero mass’ component (containing only zeros) and a count component (that may also contain zeros as well as other values), with the linked count component modelled using an appropriate count variance structure [62]. In the context of coral settlement data, we consider the zero mass component to represent the lack of availability of competent larvae whereas the count component represents the process of settlement conditional on availability of competent larvae. Because there are two model components to zero inflated models, model selection was performed by reducing the count model, then binomial model, then adding terms back to each sequentially. This process was then reversed where the binomial model was reduced, then the count model and terms added back sequentially to each. Both processes resulted in the same final model. All analyses and graphics were produced in the statistical programming language R using the PSCL and MASS packages [63].

Variation in percent live coral cover was compared among sites, and differences in the abundance of corals grouped within the same families used for the coral settlers (Acroporidae, Poritidae, ‘others’) were tested using generalized liner models (GLMs). Pocilloporiade was excluded from the analysis because the two sites with high rates of Pocilloporidae settlement (Kusu and Raffles) were also the only sites in the study where adult cover was recorded. Tukey contrast for multiple comparisons were used to identify which means contributed to any significantly differences detected. Additionally, we examined the relationship between adult coral cover and settlement rates between sites using site-specific mean coral cover and mean settlement (spat tile^-1^). We applied linear regressions separately for Acroporidae, Poritidae and ‘others’ (all other genera) over the peak settlement period (March-May 2012) and for settlement over the entire study. Due to error within response and explanatory variables (particularly the effect of high incidences of tiles with zero counts), we used a non-parametric bootstrapping approach where settlement data was resampled according to the spatial and temporal stratification of the study [64]. For analysis of the peak settlement period, only settlement tiles from Mar.–May 2012 period were resampled within each site. For total mean settlement, rather than resampling all tiles over the entire study, settlement tiles at each site were resampled within each settlement period. Each iteration of the data from coral cover transects and tile spat counts were resampled with replacement to the original sample size, within each site. A linear model was then fit to the site-specific means of the resampled data. This procedure was repeated 999 times and model coefficient estimates and other model statistics from the original model and each bootstrap iteration were stored. Bootstrap 95% confidence intervals were calculated using the percentile method [64]. If bootstrap confidence intervals for the slope included zero these models were not considered any further. Original model parameters and statistics were considered adequate if they fell within the bootstrap 95% confidence intervals.

## Results

**Fig 2:**
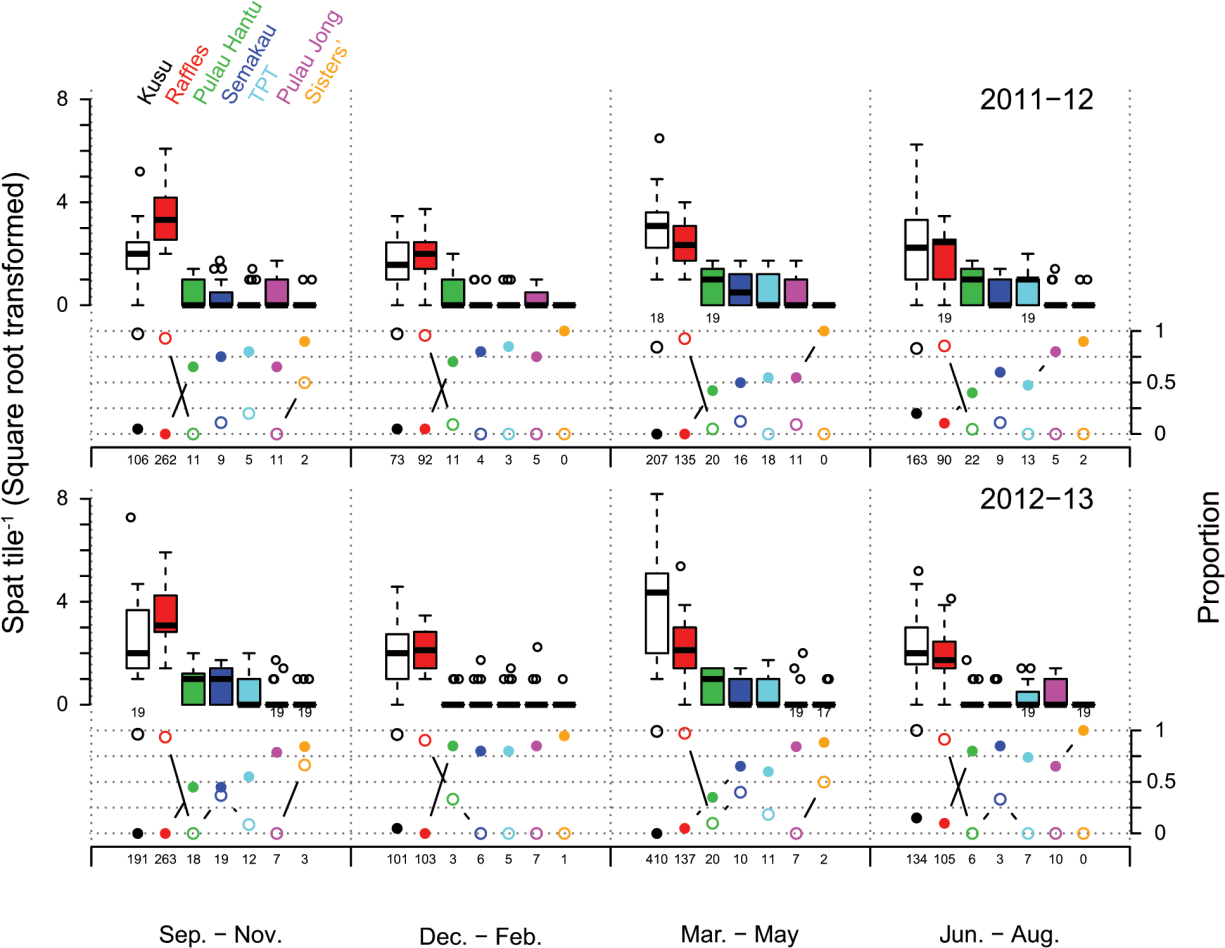
Boxplot of counts of total spat tile^-1^ (square root transformed) for each site (see legend, top left) and sampling period, for 2011–12 and 2012–13. Points above boxes represent outliers outside 1.5 × the interquartile range. Plots below boxplots show the proportion of tiles with zero spat (filled circles) and proportion of spat identified as Pocilloporidae (open circles). Twenty tiles per site, per sampling period were surveyed unless indicated under the x-axes of the boxplots. Numbers below the proportions x-axes are the total spat counts for each site and sampling period. Sites were ordered from left to right according to descending rank abundance of total spat over the entire study period.

A total of 2906 coral spat were counted on 1106 tiles over the two-year study, equating to a settlement rate of 1.31 spat tile^-1^ yr^-1^ or 54.74 spat m^-2^ yr^-1^. Coral spat settled predominately on the sides of tiles (41.7%) with fewer spat settling on the bottom (30.7%) and top (27.6%) of the tiles. Total spat counts tile^-1^ ranged between 0–67 (Fig 2). The most prominent patterns in total coral settlement were the strong and relatively consistent spatial patterns amongst sites. In any sampling period, the majority of spat observed were from two sites (Kusu and Raffles), which had close to an order of magnitude difference in total spat compared to all other sites in all sampling periods (Fig 2). Between 0–20% of tiles from Kusu and Raffles lacked coral spat (i.e. a zero count) in each sampling period, whereas values were consistently >50% for all other sites (Fig 2).

Pocilloporidae were the most frequently observed spat, accounting for ~84% of total spat, followed by taxa categorized as ‘others’ (~10%), and the families Poritidae (~4%) and Acroporidae (~1%). Damaged spat that that could not be identified taxonomically accounted for ~1% of spat. Pocilloporidae spat made up 82–100% of all spat from Kusu and Raffles in any sampling period, compared with <40% of spat from other sites, except from one site (Sisters’), during one sampling period, where two Pocilloporidae spat out of 3 total spat were observed (Fig 2). Thus, Pocillopordae spat from two of the seven sites (Kusu and Raffles) accounted for 70–90% of total spat observed in any sampling period. Due to the large site-specific differences in settlement and the constant presence of Pocilloporidae spat, spatial and temporal patterns for Pocilloporidae and the other families (Acroporidae, Poritidae, and ‘others’) were modeled separately. Pocilloporidae spat from Kusu and Raffles were examined separately, while non-Pocilloporidae spat (Acroporidae, Poritidae, and ‘others’) were examined at all sites.

### Settlement patterns of Pocilloporidae spat

The most parsimonious model of Pocilloporidae abundance at Kusu and Raffles was a negative binomial count model including temporal and spatial variation (Table 1; see S1 File for detailed model selection). A negative binomial model was necessary due to over dispersion in counts of spat tile^-1^, suggesting spatially clumped settlement at the scale of individual tiles. This pattern was most apparent at Kusu, where for example, in a single season (Mar.–May 2012) the rank bottom 30% of tiles contained 1–4 spat each, compared with 25–67 spat for the rank top 30% of tiles.

**Table 1:** Summary of final model results of Pocilloporidae spat settlement at Kusu and Raffles Lighthouse, evaluated by likelihood ratio tests of nested models.

Term Dropped	LogL	∆LogL	Df	∆df	*X* ^*2*^	*p*
Full Model	-936.43		10			
Year	-939.34	-2.91	9	-1	5.8113	0.01592
Site × Season	-949.18	-12.75	7	-3	25.482	<0.0001

Final model was a negative binomial generalized linear model with a Year effect and Site × Season interaction.

**Fig 3:**
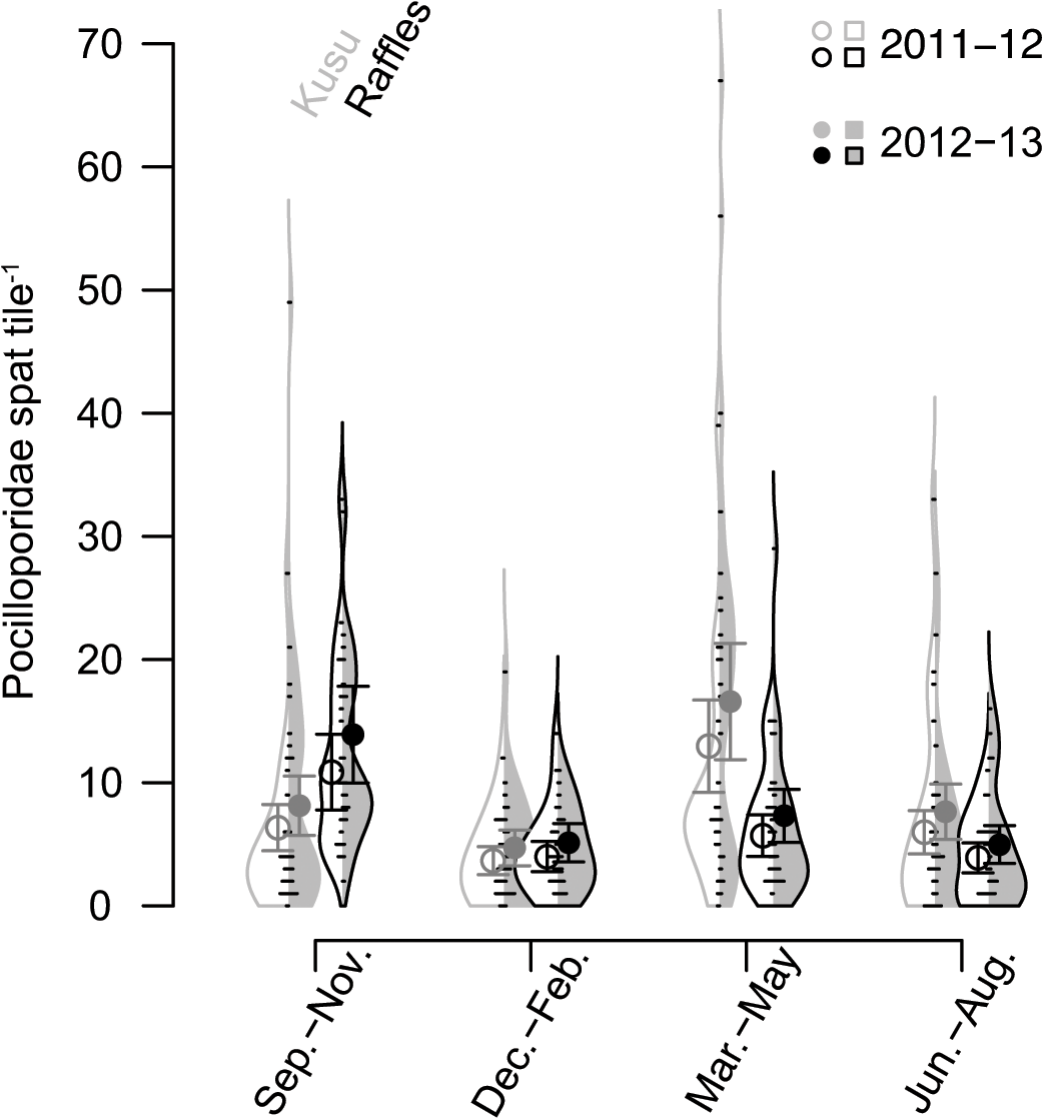
Beanplot of Pocilloporidae settlement at Kusu (grey outlines and points) and Raffles Lighthouse (black outlines and points). Shape of vertical distribution represents relative smoothed density distribution of counts. White fill on left side of beans represents 2011–12, and grey fill on the right side of beans represents 2012–13. Points are predictions from final model ± 95% CI.

There was a clear effect of year with more settlement occurring during Sep.–Aug. 2011–12 than Sep.–Aug. 2012–13, however differences between sampling periods within years were confounded spatially, with different seasonal peaks in settlement at the two sites (Fig 3). Accounting for yearly variation, peak settlement occurred in Mar.–May at Kusu and Sep.–Nov. at Raffles, with estimated spat tile^-1^ in the peak season around double that of any other season in each year at each site (Fig 3). Settlement was consistent outside of the peak season at both sites and similar to non-pocilloporid settlement with the lowest settlement occurring between Dec.–Feb. at both sites.

### Settlement patterns of non-Pocilloporidae spat

Given the large number of zero counts in the data, zero-inflated count models were used to assess the importance of spatial and temporal predictor variables. The most parsimonious model of non-Pocilloporidae abundance was a zero inflated negative binomial model including temporal and spatial variation (Table 2; see S1 File for detailed model selection). There were spatial (site) differences in both the count and zero-mass (binomial) model components and temporal variation in the zero-mass component (Table 2). A negative binomial count model was necessary due to over dispersion in counts of spat tile^-1^, suggesting spatially clumped settlement at the scale of individual tiles when settlement occurred.

**Table 2:** Summary of final model results of non-Pocilloporidae spat settlement evaluated by likelihood ratio tests of nested models.

Term Dropped	LogL	∆LogL	Df	∆df	*X* ^*2*^	*p*
Full Count Model	-815.39		22			
Site	-841.85	-26.46	16	-6	52.913	<0.0001
Full Binomial Model	-815.39		22			
Season × Year	-833.13	-17.74	19	-3	35.477	<0.0001
Site	-847.17	-32.43	16	-6	64.858	<0.0001

Final model was a negative binomial zero inflated model with the Site term in the count component and Site, Season, Year and Season × Year interaction in the zero mass binomial component.

**Fig 4:**
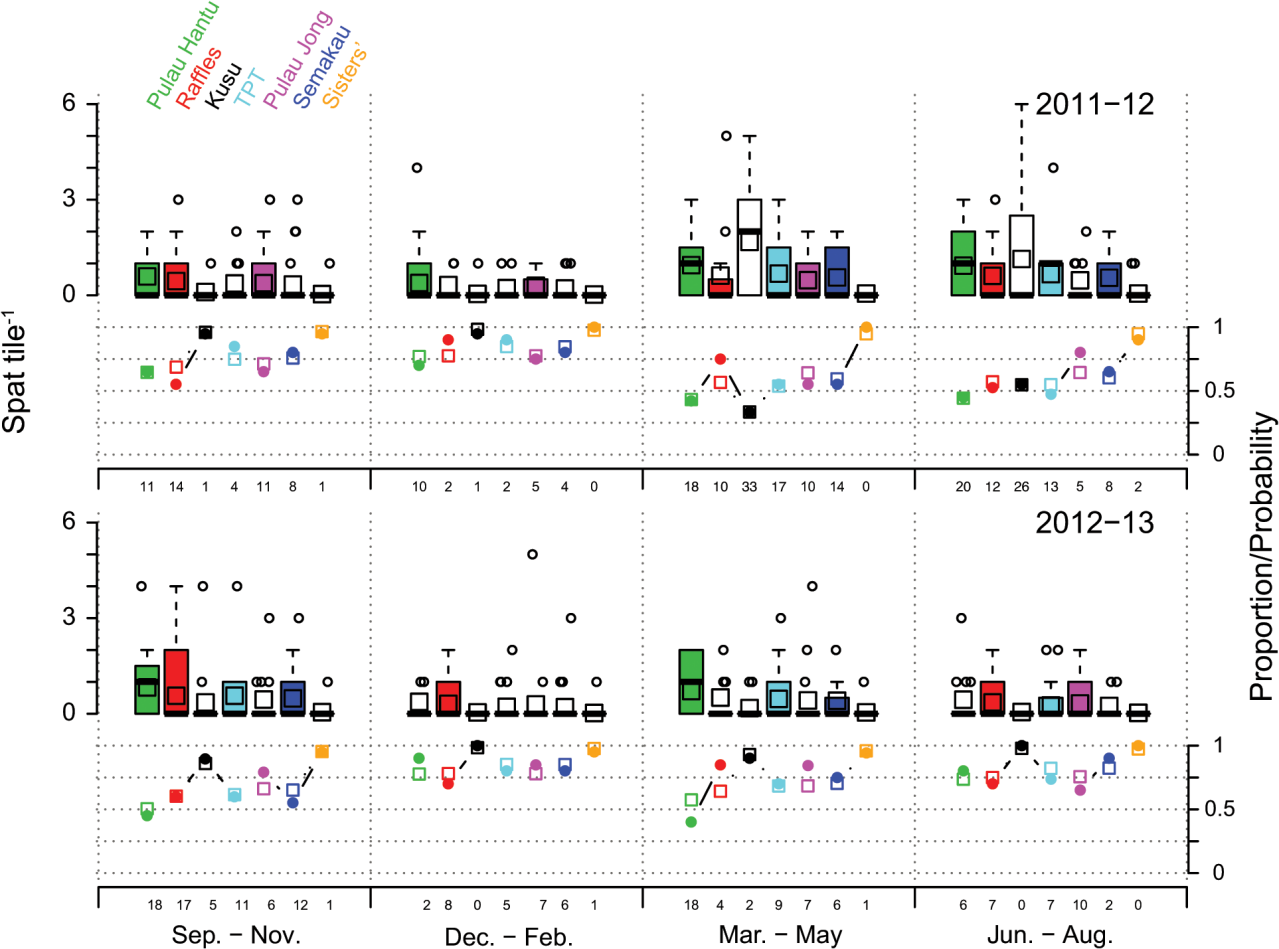
Boxplot of non-Pocilloporidae spat tile^-1^ for each site (see legend, top left) and sampling period for 2011–12 and 2012–13. Points above boxes represent outliers outside 1.5 × the interquartile range. Plots below boxplots show the proportion of tiles with zero spat (open circles). Open squares on boxplots and line plot show the predicted values from the final zero-inflated negative binomial model predicted spat tile^**-1**^ and predicted probability of a zero count, respectively. Numbers below the proportion/probability x-axes are the total non-Pocilloporidae spat counts for each site and sampling period. Sites were ordered from left to right according to descending rank abundance of non- Pocilloporidae spat over the entire study period.

Spatial (site) differences in zero mass and count were due to the consistently relatively high zero mass and low counts at two sites (Kusu and Sisters), and how this changed temporally for Kusu, compared with relative changes at all other sites. Kusu and Sisters shared the highest and second highest proportion of zeros and lowest estimated spat tile^-1^, in six of eight sampling periods (Fig 4). In the remaining two sampling periods (Mar.–May and Jun.–Aug. 2012), Sisters maintained the highest proportion of zeros and lowest spat tile^-1^, whereas Kusu changed to having a relatively low zero mass, the highest total spat count and highest estimated spat tile^-1^ of all sites (Fig 4). Sites apart from Kusu and Sisters had similar spat tile^-1^ relative to each other within each sampling period, with spat tile^-1^ at Sisters lower than all other sites in all sampling periods (Fig 4).

Accounting for spatial variation between sites, the zero-mass varied temporally between sampling periods, within and between years (Table 2), indicating the availability of competent larvae differed at these temporal scales. Peak abundance of Non-pocilloporidae coral spat differed between the two study years (Fig 4). In the first year (September 2011–August 2012), the lowest site-specific probability of a zero count was from Mar.–May, with marginally lower total settlement rates occurring from Jun.–Aug. (Fig 4). In contrast, the second year (September 2012–August 2013) had the lowest probability of a zero count and highest settlement from Sep.–Nov. (Fig 4). In both years the least settlement occurred at the end of the wet season during the Northeast monsoon (Dec.–Feb.), with both years displaying a similarly low spat abundance during this period. The Jun.–Aug. 2012 period had lower, but similar spat abundance to Mar.–May 2012, such that the three sampling periods displaying the lowest probability of a zero count and highest spat abundance were contiguous sampling periods spanning 9 months (Mar.–Nov. 2012), over the first inter-monsoon, the southwest monsoon, the second inter-monsoon and the start of the northeast monsoon (Fig 4).

### Local coral assemblage structure

**Fig 5:**
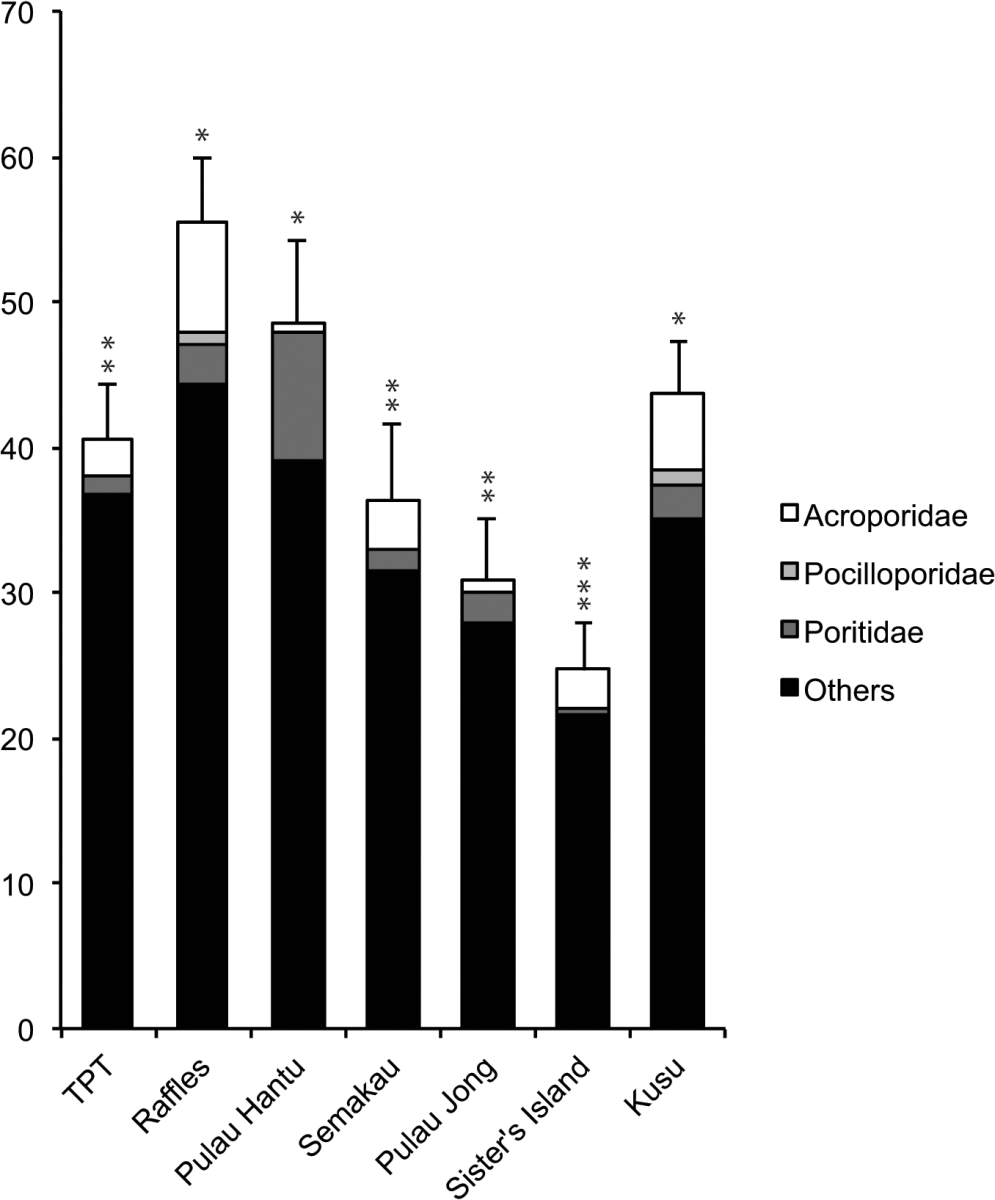
Percent coral cover at each site for Acroporidae, Pocilloporidae, Poritidae and ‘others’. Data are means ± SE, and asterisks (*) indicate significant differences among sites (Tukey’s comparison test, p <0.05). Sites arranged from west to east (see Fig 1).

Coral cover differed significantly among sites (*X* ^*2*^ = 57.53, df = 6,41, p < 0.0001), with the highest coral cover at Raffles (55.4 ± 4.4%) and lowest at Sister’s Island (24 ± 3.6%; Fig 5). The most common genera were *Pectinia* (13.8 ± 1.5%), *Merulina* (13.0 ± 1.6%), *Pachyseris* (11.3 ± 1.6%), *Montipora* (6.6 ± 2.1%), *Echinopora* (6.6 ± 1.1%) and *Platygyra* (6.3 ± 0.9%). Collectively, these six genera accounted for 58% of total coral cover across all sites. Percent cover of coral families and ‘others’ related to settled spat differed significantly among sites: Acroporidae (*X* ^*2*^ = 39.66, df = 6,41, p < 0.0001); Poritidae (*X* ^*2*^ = 46.16, df = 6,41, p < 0.0001); and ‘others’ (*X* ^*2*^ = 57.52, df = 6,41, p < 0.0001). Percent cover of Acroporidae was significantly higher on Raffles (7.7 ± 1.7%) and Kusu (4.6 ± 1.4%) compared to all other sites, which had <3% cover. Percent cover of Poritidae was highest on Pulau Hantu (8.7 ± 4.1%) while Pocilloporidae was recorded on only two sites (Kusu and Raffles; Fig 5).

**Fig 6:**
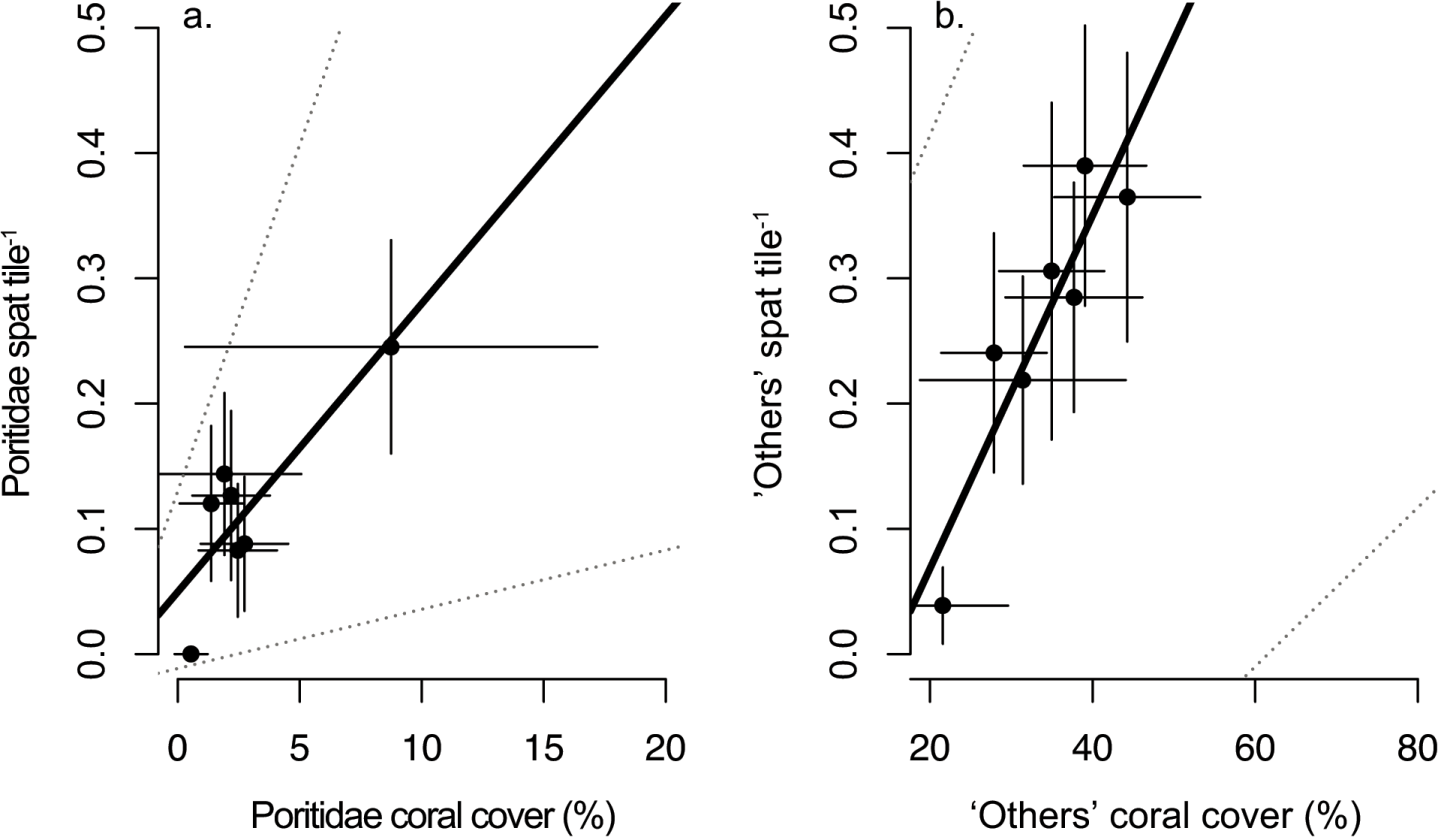
The relationship between adult coral cover and mean settlement over the entire study for a.) Poritidae and b.) ‘others’. Error bars represent standard error. Dotted lines represent 95% confidence intervals from bootstrapped regression parameters.

There was no relationship between percent coral cover and mean settlement tile^-1^ during the peak settlement period for any coral family (Acroporidae: *F* = 0.09, *df* = 1.5, *p* = 0.771; Poritidae: *F* = 0.0005, *df* = 1.5, *p* = 0.983) or ‘others’ (*F* = 3.22, *df* = 1.5, *p* = 0.132). However, there were statically significant relationships between adult coral cover and settlement tile^-1^ over the entire study period for Poritids (R^2^_adj_ = 0.64, *F* = 11.81, *df* = 1.5, *p* = 0.018, Fig 6A) and ‘others’ (R^2^_adj_ = 0.80, *F* = 25.86, *df* = 1.5, *p* = 0.038, Fig 6B), but not for Acroporidae (*F* = 0.0004, *df* = 1.5, *p* = 0.98).

## Discussion

Coral settlement in Singapore showed a high degree of spatio-temporal variation consistent with patterns reported from other Indo-Pacific reefs at a range of latitudes [38,39,64]. Overall, rates of coral settlement in Singapore were low (54.74 spat m^-2^ yr^-1^) compared to other low latitude Indo-Pacific locations, including: Seychelles–595 spat m^-2^ yr^-1^ [65] and Indonesia–527 m^-2^ yr^-1^ [66], but see [67], however well above previously reported values for Singapore of 14.7 spat m^-2^ yr^-1^ [54]. Spat settlement occurred year round, but peak settlement periods were apparent. For Pocilloporidae, peak settlement of coral spat occurred between March–May at Kusu and between September–November at Raffles in both study years. In contrast, for non-Pocilloporidae species, peak settlement occurred between March–May in 2012, but settlement remained high through to September–November 2012. Peak settlement of non-Pocilloporidae species was consistent with the predicted periods for broadcast coral spawning (March–April and October: [58,59,68]) occurring during the inter-monsoon periods, while the lowest settlement occurred from December–February during the northeast monsoon.

Temporal patterns in coral settlement generally match those predicted on the basis of known reproductive patterns and life histories. For most broadcast spawning corals species, larval settlement is highly seasonal [69] because they have a single annual gametogenic cycle followed by synchronous spawning [70]. For example, on the Great Barrier Reef (GBR), the settlement of many broadcast-spawning genera (e.g. *Acropora*) peaks during the months coinciding with peak reproductive season from October to November [70]. Similarly, most broadcast spawning coral species in Singapore (e.g., Acroporidae, Poritidae and Faviidae) also exhibit marked seasonal reproduction with major spawning occurring after the full moon in March or April [58,59], therefore highly seasonal coral settlement patterns were expected. However, peak settlement for non-Pocilloporidae broadcast spawning species varied within and between years. For example, peak settlement was recorded in March–May 2012 with mean settlement rates of 0.74 spat tile^-1^ and this coincided with the main coral spawning period that year [71]. In contrast, the mean settlement rates during the March–May 2013 were 0.34 spat tile^-1^, >2 times lower. Inter-annual variation in settlement rates has been widely documented (e.g., [23,69]), and often reflects variation in availability of competent larvae in response to reproductive success or prevailing meteorological, climatic, or hydrodynamic conditions around the time of spawning [19,72]. One possible explanation for lower settlement in 2013 is that a split spawning (*i*.*e*., when the full moon occurs late in the spawning month leading to coral populations dividing spawning over consecutive months: [73]) led to lower levels of synchrony and hence lower settlement rates (e.g. [19]).

More interesting, however, was the extended period of high settlement outside the predicted spawning period from June to November 2012 (Fig 4). Similar periods of extended high coral settlement have been observed on the central GBR [74], thus this occurrence is not restricted to equatorial locations. For example, over a two-year study investigating coral settlement patterns on patch reefs in the lagoon of Walker Reef, Baird et al. [74] noted a six-month period of high settlement from October to March. These authors suggested that the extended coral settlement was likely the result of either larvae produced by colonies spawning later in the season or long-lived larvae. In Singapore mature gametes have been found in coral colonies in at least 6 months (March–May and September–November) for a range of species [58,59,68,75]. For example, up to 20% of sampled colonies of *Porites lutea* and *Platygyra pini* contained mature oocytes between September and November [58,75], and some colonies of *Acropora humilis* contained mature eggs both in October/November and April indicating bi-annual spawning [58]. Similarly, *Hydnophora exesa*, *Merulina ampliata* and *Echinopora lamellosa* were all found to contain mature oocytes (stage IV) in both April and October [68]. Nonetheless, this does not explain the influx of coral spat onto Singapore reefs between June and August. Although previously examined species represent some of the most common species found on Singapore’s reefs, it is plausible that at least some species may spawn outside of the main spawning season, which would contribute to an extended period of coral settlement. Of the 255 species recorded in Singapore [52] only about 50 coral species (or 20%) have been sufficiently examined to determine when they spawn [58,59,68,75].

An alternative explanation for an extended settlement period is the length of the larval phase. Available data from a few coral species in Singapore indicates peak settlement 2 to 6 days post fertilization [76]. However coral larvae from many species can survive in water column for periods exceeding 100 days [21], allowing substantial dispersal potential [22]. Consequently, coral spat observed from June to August could have conceivably originated from colonies that spawned during March, April or May. Furthermore, it is also possible that the influx of coral spat during this period originated from coral populations outside Singapore, where corals spawn at slightly different times. Some coral species in Indonesia, for example, have been documented spawning between January and November (e.g. [77]).

In contrast to broadcast spawners, the larval settlement of many brooding species (e.g. *Pocillopora damicornis*) is generally less seasonal and year round, corresponding to the longer period over which planulae are released [74,78]. In Singapore, *Pocillopora damicornis* is the most common known brooding species of the family Pocilloporidae [52] and planulates larvae monthly [55]. While it is possible that some Pocilloporidae spat originated from other species or from nearby reefs outside of Singapore, considering that Pocilloporid spat were abundant at only two sites where *P*. *damicornis* adults are present (i.e. Kusu and Raffles) it is likely that the majority of spat counted in this study were from *P*. *damicornis*. Settlement of Pocilloporidae spat in Singapore was year round but highly seasonal suggesting that the reproductive output of these species likely peaks in both March–May and September–November. Importantly, this suggests that even in equatorial locations, with relatively low seasonal environmental variation, there may be periods of the year that are more favorable for larval release, settlement and survival [67]. Moreover, given that the larvae of brooding species are typically ready to settle on release, whereas broadcast spawning species have an obligate planktonic period (i.e. 3–14 days: [22]), pulses in settlement in brooding species are more likely to be affected by local hydrodynamic and environmental conditions prevailing at the time of release (i.e., neap tide, slack water) which aggregate more larvae. Peak settlement of Pocilloporidae occurred during the inter-monsoon periods, which are characterized by having the lowest annual net transport changes [57]. This may partially explain the greater retention of larvae during these periods.

Apart from the temporal patterns, results also showed strong, consistent spatial heterogeneity in coral settlement with marked variation in the abundance of coral spat among the seven sites. Overall coral settlement (i.e. non-Pocilloporidae and Pocilloporidae spat) was highest on Kusu and Raffles compared to all other sites during all periods, while Sister’s Island had the lowest settlement in all periods. Most interesting, was the order of magnitude higher numbers of pocilloporid spat at Kusu and Raffles compared to all sites, and its recurrence among all sampling seasons, suggesting that this is a product of natural systematic processes rather than chance events (Figs 2 and 3). Consistencies in spatial patterns of pocilloporid settlement between years have also been documented in the Red Sea [79] and on the Great Barrier Reef [80]. Sites that consistently receive higher levels of settlement, known as ‘recruitment hotspots’ (*sensu* [80]), may be crucial to the persistence of populations [78]. Recent studies suggest that a combination of deterministic and stochastic processes likely influence settlement variation at these scales [80]. For example, on One Tree Island, Eagle et al. [80] detected ‘recruitment hotspots’ in the lagoon and on the reef slope for the families Pocilliporidae, Poritidae and Acroporidae and these were associated with differences in hydrodynamics (i.e., water flow) and/or the abundance of adult conspecifics. Recent hydrodynamic flow models developed for Singapore predict higher larval settlement onto Raffles and Kusu than other reefs in Singapore (Set I simulations: [81]). Furthermore, Kusu and Raffles were the only sites with adult Pocillopora colonies recorded during the surveys. Adult coral cover of Pocillopora on Kusu and Raffles is likely driven by strong local retention of brooded larvae capable of immediate settlement on release. Further work is required to determine the specific mechanisms behind the patterns of *Pocillopora* settlement observed, and hence the distribution of adults

The composition of coral spat in Singapore contrasts markedly with studies on tropical reefs in other parts of the Indo-Pacific, including: the GBR [23,74] and Palau [82] because there were few Acroporidae (<1%) and many Pocilloporidae spat (84%). However, the composition of spat in Singapore was similar to other equatorial reefs in Kenya [39], Seychelles [65], and some near-shore reefs in Indonesia [66,67] where the dominance of Pocilloporidae was also reportedly high (60–90% of total spat). The most abundant non-Pocilloporiade spat were from families other than Acroporidae and Poritidae (i.e. ‘others’). These settlement patterns are readily explainable by the adult assemblage structure in Singapore, which is dominated by sediment-tolerant coral taxa [52]. Families other than Acroporidae and Poritidae accounted for 84% of the total coral cover. Of these families, five coral genera (*Merulina*, *Pachyseris*, *Platygyra*, *Pectinia*, and *Echinopora*) accounted for nearly 50% of the cover, whereas both Acroporidae and Poritidae accounted for <8%, respectively.

There were also marked differences in the composition of coral spat and the corresponding abundance of adult corals among sites in Singapore. Pocilloporidae settled in a disproportionate abundance compared to other families in two sites. *Pocillopora* is considered an opportunistic genus, capable of high recruitment, but also presenting high turnover and mortality [83]. Results indicated no relationships between adult cover and settlement during the peak spawning period for any other coral family, but apparent positive relationships exists for Poritidae and ‘others’ and adult cover over the entire study period, indicating local abundance is potentially driving local patterns in larval supply. However, these results require cautious interpretation because of the wide range bootstrap estimates and confidence intervals. Moreover, we did not measure fecundity simultaneously with rates of settlement [19] or assess the importance of post-settlement processes [24]. Penin et al. [24], for example, found that adult colonies and juvenile corals (1–5 cm diameter) were positively correlated in French Polynesia, but found no relationship between adults and recently settled spat (≤3 mo old), and implicated fish grazing as the source of post-settlement mortality. Newly settled corals often experience very high rates of mortality with up to 99% of individuals dying within the first few months [84,85], due to overgrowth by macroalgae, competition with conspecifics or other benthic organisms, and predation (see review by [86]). Settlement and early post settlement processes are also greatly influenced by anthropogenic impacts such as high sedimentation, which can reduce substrate availability and smother recently settled spat, and high turbidity, which reduces light and subsequent growth (see review by [31]). Considering both high sedimentation and high levels of suspended sediment reported for Singapore [45,46], it is likely that these processes are influencing rates of settlement and potentially disrupting post settlement processes on Singapore’s reefs, and therefore require further examination.

Low rates of settlement in Singapore may also indicate that processes other than recruitment alone, such as regrowth of remnant colonies and fragments (e.g. [17,87]) or asexual reproduction (e.g. [88]), play an equally important role in population maintenance of these reef communities. For example, Gilmour et al. [17] showed that the recovery of heavily impacted coral assemblages on an isolated reef system in Western Australia (i.e., Scott Reef) were the result of high growth rates and survival of remnant colonies (mainly *Acropora* spp.) prior to rapid increases in juvenile recruitment as colonies matured. Alternatively, asexual reproduction by coral fragmentation, considered an adaptation to unfavorable local environmental conditions [89], may allow coral species to persist when they are unable to complete their full sexual reproductive life cycle (i.e. recruitment; [88]). Although asexual fragmentation is more common among branching coral species (e.g. *Acropora*; [18]), Foster et al. [88] recently reported that massive coral species (e.g. *Montastraea annularis*) are also capable of propagating using asexual methods. Given the sustained anthropogenic disturbances occurring on Singapore’s reefs, which may reduce the success of sexual recruitment, further research on the importance of other processes involved in maintaining coral cover and recovery from disturbances are warranted.

## Conclusions

In summary, coral settlement rates in Singapore are low compared to other equatorial regions, but revealed periods of peak settlement despite year round settlement and sustained anthropogenic disturbances (i.e. high sedimentation and turbidity). The composition of coral spat is greatly overrepresented by locally brooded Pocilloporidae spat from two sites, suggesting that sexual recruitment from other coral families is being negatively affected. Furthermore, current patterns of settlement are reinforcing the local adult assemblage structure (‘others’; i.e. sediment-tolerant coral taxa) in Singapore. Consequently, the replenishment capacity of Singapore’s reefs appears relatively constrained, which could lead to less resilient reefs. However, the persistence of coral assemblages in Singapore suggests that other ecological processes in addition to sexual recruitment (e.g. rapid regrowth of remnant corals or asexual reproduction) may also play an important role in population maintenance of these reef communities. Further studies are necessary to elucidate mechanisms that regulate early life history processes (i.e. settlement and post-settlement mortality) and whether regrowth of remnant corals or asexual reproduction are important processes for maintaining coral populations on highly urbanized reef systems.

## Supporting Information

S1 File Detailed model selections for Pocilloporidae spat (Kusu and Raffles) and non-Pocilloporidae (all sites). (XLSX)
